# Management of cervical spine trauma in children

**DOI:** 10.1007/s00068-018-0992-x

**Published:** 2018-08-24

**Authors:** Phillip Correia Copley, Vicky Tilliridou, Andrew Kirby, Jeremy Jones, Jothy Kandasamy

**Affiliations:** 1 Department of Neurosurgery, Western General Hospital, University of Edinburgh, Crewe Road South, Edinburgh, EH4 2XU UK; 2 Department of Radiology, Royal Infirmary of Edinburgh, Edinburgh, UK; 3 Department of Radiology, The Royal Hospital for Sick Children, Edinburgh, UK

**Keywords:** Cervical spine, Children, Pediatric, Trauma, Fracture, SCIWORA

## Abstract

**Purpose:**

Paediatric cervical spine injuries are fortunately a rare entity. However, they do have the potential for devastating neurological sequelae with lifelong impact on the patient and their family. Thus, management ought to be exceptional from the initial evaluation at the scene of the injury, through to definitive management and rehabilitation.

**Methods:**

We set out to review cervical spine injuries in children and advise on current best practice with regards to management.

**Results:**

Epidemiology, initial management at the scene of injury, radiological findings and pitfalls of cervical spine trauma are outlined. Strategies for conservative and surgical management are detailed depending on the pattern of injury. The management of spinal cord injuries without radiological abnormality (SCIWORA) and cranio-cervical arterial injuries is also reviewed.

**Conclusions:**

Due to a paucity of evidence in these rare conditions, expert opinion is necessary to guide best practice management and to ensure the best chance of a good outcome for the injured child.

## Introduction

Although cervical spine trauma in children is fortunately relatively infrequent, the potential biopsychosocial effects on the individual, family and wider society may be huge. Therefore, a good understanding of the underlying mechanisms, characteristic patterns and differences between managing adults and children with these potentially devastating injuries is imperative if management is to be effective.

## Epidemiology

One of the largest epidemiological studies of trauma in children in the USA assessed the National Pediatric Trauma Registry over a consecutive 10-year period and identified that from 75,172 injured children, only 1.5% had a cervical spine injury (1098 patients) [1]. The study found that upper cervical spine injuries were prevalent among all age groups (42% in those aged ≤ 8 years; 58%, aged > 8 years), whereas lower cervical spine injuries were more common in older children (85% occurring in those aged > 8 years) [1]. Indeed, younger children often sustain injuries in the upper portion of the cervical spine because the relative size of the head compared to the rest of the body is greater in this age group. As the child grows, this pattern evolves into that more commonly seen in adults, where injury to the lower cervical spine is far more common. Mortality was 33% in those with an upper cervical spine injury, versus 8.3% in lower cervical spine injury [1]. As with adults, males show a greater propensity to suffering cervical spine injury than females, at a ratio of 1.5–2.1:1 [2].

Blunt trauma is the commonest cause of cervical spine injury, attributing to 95% of injuries, commonly as a result of motor vehicle accidents (61%). Of these incidents, 68.9% were passengers in the motor vehicle, 22.9% were pedestrians and 8.2% were bicycling when the accident occurred [1]. Falls were a greater cause of injury in the younger group (18% in those aged ≤ 8 years; 11%, aged > 8 years) [1]. Sports related injuries were far more common in the older group (3% in those aged ≤ 8 years; 20%, aged > 8 years) [1]. The possibility of non-accidental injury should always be considered by the attending clinician, especially in the younger age group.

In neonates, it is estimated that one in 60,000 births are complicated by spinal cord injury [3]. Maternal risk factors include a small pelvis, obesity, diabetes and primiparity [4]. Risk factors related to the foetus include abnormal position of the neonate during delivery, prolonged delivery, the use of forceps to aid delivery and shoulder dystocia. Brachial plexus injury is far more common than spinal injury and can affect the upper (C5/6) or lower (C7/T1) nerves causing Erb’s or Klumpke’s palsy, respectively. Although fortunately rare, damage to the cervical spine itself can lead to respiratory compromise (due to paralysis of the respiratory musculature and/or phrenic nerve injury) and hypotonia with a flaccid paraplegia or quadriplegia [4]. Indeed, this may prove fatal [5].

## Anatomical differences in children

One of the most important differences between the vertebral column of children and adults is its intrinsic elastic properties. This elasticity may cause immediate self-reduction following dislocation during injury. This may be partly responsible for the often-normal bony alignment when radiography and computed tomography (CT) are performed upon initial work-up. In fact, children are more likely to suffer ligamentous damage than adults and the syndrome of spinal cord injury without radiological abnormality (SCIWORA) has been coined to classify these types of non-bony injuries where no injury is found on X Ray/CT scans [6]. Indeed, spinal cord injury occurs in approximately 35% of cervical spine injury and approximately half of these cases demonstrate no radiological sign of bony injury [1].

The inherent elasticity of the cervical spine is, in part, due to the shallow and horizontally oriented facet joints which allow increased range of flexion and extension. Moreover, ligaments can stretch farther without suffering damage. Hook-shaped uncinate processes from C3 to T1 act to prevent spondylolisthesis and limit lateral flexion in the adult. However, these are underdeveloped in the child and so render the cervical spine more flexible. Supporting musculature is also underdeveloped and this may represent another important factor. The larger head in relation to the body acts to increase moment force through the upper cervical spine and increases the risk of injury at the fulcrum, which lies at C2–3 in infants. As the child grows, the fulcrum descends to the adult position at C5–6 [7].

## Initial management principles

The Advanced Trauma Life Support (ATLS**®**) principles apply to immediate management of a child with cervical spinal injury. Immobilisation in a neutral position should be performed at the scene of the accident and this is maintained until the child can be fully assessed in the emergency department. Immobilisation prevents the scenario in which instability is initially overlooked and neurological deficit progresses during transfer of the child as a result. Traditionally, as per adults, children with suspected cervical spine injuries have been placed on a spinal board and a rigid cervical collar applied, with sandbags or blocks either side of the head and tape to secure and immobilise the head [8]. However, rigid collars often fit children poorly, especially in the very young. Moreover, children may be agitated and in pain, making application of the collar difficult and potentially dangerous. The safest approach is a pragmatic one, allowing the child to find a comfortable position and providing manual in-line stabilisation initially. If a properly sized collar can be safely fitted, then this is appropriate at this stage; if not, then simply maintaining a neutral or comfortable position with blocks/rolled up towels placed either side of the head and tape to secure them in place is appropriate. This approach has been advocated by NICE and the advanced paediatric life support course [9, 10]. It is important to also be aware that a cervical collar may potentiate atlanto-occipital distraction and worsen neurological injury in such cases. Therefore, if this pattern of injury is suspected then sandbags and tape only should be used [11]. As the young child’s head is proportionally much larger in comparison to his/her body when compared to an adult, when lying flat on a spinal board the head is forced into a slight degree of flexion. Therefore, an occipital recess or thoracic elevation may be necessary to counteract this effect [12].

Damage to the cervical spine should be considered in the presence of suspicious injury, unconsciousness, torticollis, rigidity of the cervical musculature, significant neck pain, or neurological signs/symptoms that may be either transient or permanent (radiculopathy/myelopathy depending on pattern of injury). Children are less likely to suffer from neurological injury than adults in the presence of trauma, and incomplete spinal cord injuries (SCI) are more common (75% incomplete versus 25% complete) [1]. Complete spinal cord injury, with loss of motor and sensory function below the level of injury, is often irreversible and leads to devastating consequences.

If a spinal cord injury is present, damage is termed primary or secondary. Primary injury is sustained at the time of trauma due to mechanical insult and is irreversible. Secondary injury is sustained after the incident and one of the greatest contributors to this is cord hypoperfusion. Thus, preventing hypotension to prevent cord ischaemia is paramount during the resuscitation phase. Appropriate organ support in an intensive/high dependency care setting is mandatory in the subsequent phase of management. A detailed examination must be performed so that patients with SCI can be stratified as per the American Spinal Injury Association (ASIA) impairment scale to properly determine the extent of the injury (http://asia-spinalinjury.org/wp-content/uploads/2016/02/International_Stds_Diagram_Worksheet.pdf).

Neuroprotective therapies have been sought in an attempt to prevent secondary damage in acute SCI, which is thought to be (in part) caused by cord oedema and inflammatory processes consequent to the initial injury. The use of methylprednisolone has been particularly controversial. It was popularised following the publication of the National Acute Spinal Cord Injury Surveys (NASCIS) II and III in the 1990s, which showed a beneficial effect in SCI if given within 8 h of injury [13, 14]. However, these studies only assessed patients over 13 years of age. Moreover, since publication of this data, re-analysis and other studies have demonstrated that the adverse effects of steroids may outweigh any benefit [15]. Based on this, the Congress of Neurological Surgeons changed their stance on this subject in their most recent guidelines (2013), stating that methylprednisolone should not be used routinely in spinal cord injuries [16]. Other authors have challenged this stance, stating that the current evidence available can be used neither recommend, nor to refute the use of methylprednisolone [17]. They state that the decision should be made on a case-by-case basis, and advocate that methylprednisolone may be especially effective in cervical spinal cord injuries undergoing decompression [17]. This ongoing controversy highlights the need for further high quality randomised control trials to further assess the clinical use of steroids in spinal cord injuries. GM-1 ganglioside, an anti-excitotoxic compound, was another drug that showed promise in early studies; however, a large multicentre randomised control trial showed no difference in neurological outcome and so again, current guidelines do not recommend its use [16, 18]. Hypothermia has also been used to mitigate the effects of inflammation on the spinal cord with promising improvements in neurological outcome in animal and small human studies. Nevertheless, there remains a need for high quality evidence to determine whether the benefits will outweigh the risks of this therapy [19]. Further research continues desperately to elucidate a potential therapy that might improve the long-term prognosis in spinal cord injuries.

With regard to surgical intervention, decompression attempts to reduce secondary damage following SCI that is attributable to oedema and ischaemia. Experimental studies have shown beneficial effects in outcomes of in vivo animal models of SCI. Decompression is usually performed alongside stabilisation in patients with incomplete SCIs with extrinsic compression. The timing of surgical intervention is controversial, with a paucity of level I evidence. Whilst theoretical and experimental animal studies suggest early intervention would benefit, clinical studies have yet to prove this. Current expert opinion suggests the optimal time-frame for operative intervention is 8–24 h [20]. Such early intervention allows shorter periods of hospitalisation and earlier mobilisation, which may reduce the morbidity associated with prolonged bed-rest and hastens the commencement of post-injury rehabilitation.

## Clearing the cervical spine and principles of imaging

### Plain radiography

Vicellio et al. performed a prospective study assessing over 3000 children with cervical spine injury using the NEXUS criteria (Table 1) that had previously been established for use in adults for clinical clearance of the potentially injured cervical spine [21, 22]. All children received three-view radiography. It was identified that if the child did not exhibit any of the five NEXUS criteria, then they did not have a cervical spine fracture [21]. However, only 2.8% of children in this study were under 2 years old and this group are the most difficult to assess in the emergency department [2].

**Table 1:** NEXUS 5 point criteria [21, 22]

Midline tenderness
Intoxication
Altered level of alertness (inc. intubation)
Focal neurological deficit
Painful distracting injury

The congress of neurological surgeons [23] advise that there is level II evidence stating that children older than 3 years should not be imaged if: alert, neurologically intact, without posterior midline cervical tenderness (with no distracting pain), not hypotensive without explanation and are not intoxicated. In children younger than three, Level II evidence states that children should not be imaged if: GCS > 13, neurologically intact, without posterior midline cervical tenderness (with no distracting pain), not hypotensive without explanation, are not intoxicated and were not confirmed/suspected to have been involved in one of the following scenarios (motor vehicle accident, fall > 10 feet, non-accidental trauma). If these criteria are not all met then radiographic or CT imaging of the cervical spine is necessary.

Controversy remains as to which is the best imaging modality in the context of trauma to the cervical spine. Whilst cervical radiography is not as sensitive as CT imaging, it delivers a much lower dose of radiation and so offers a safer initial test. Garton & Hammer applied the NEXUS criteria to 190 children treated with cervical spine injury, and determined 75% sensitivity in children younger than 8 and 93% sensitivity in those older than eight with plain radiography [24]. Another study showed that antero-posterior and lateral radiography have a 87% sensitivity for children less than 9 years of age, and addition of the odontoid peg view (which is often difficult to achieve technically) does not aid in diagnosis [25].

Flexion–extension radiography helps to determine the stability of the cervical spine. However, these cannot be performed in children with acute neurological deficits and are not feasible in children with cervical muscle spasm post-injury. Therefore, they play little role in the acute setting and a reserved for assessing patients on follow-up after treatment of cervical spine injures to assess for delayed instability. During follow-up of cervical spine injuries, flexion/extension radiography can assist the decision making as to whether it is safe to start to wean the patient off of an external orthosis. Imaging should only be performed by trained radiographic practitioners and only on the request of a spinal surgeon.

### Computed tomography (CT)

In Garton & Hammer’s study, CT exhibited a 94 and a 97% sensitivity in children younger/older than eight years, respectively [24]. Whilst CT scanning also has a much better specificity for identifying cervical spine injuries, many clinicians are rightly cautious in their use of CT scanning in the paediatric population due to the long-term risks of ionising radiation [26]. Indeed, the congress of neurological surgeons [23] advises that the only level I evidence in children is that any patient with potential atlanto-occipital dislocation requires a CT scan. Otherwise, all other recommendations are based on level II–IV evidence.

The National Institute for Health and Care Excellence (NICE) have advised CT scanning of the cervical spine within 1 h of identification of one of the following risk factors: GCS < 13 on admission, if intubated, focal neurological signs, paraesthesia in the upper/lower limbs, if there is strong clinical suspicion of injury despite normal radiographs, if radiography is technically difficult/inadequate or if radiographs identify a significant bony injury [27]. NICE also advise CT if a definitive diagnosis of cervical spine injury is needed urgently (for example, before surgery) [27]. If these factors are not identified, radiography should be performed within 1 h if safe assessment of range of neck movement cannot be made or a dangerous mechanism of injury is present (fall from a height of greater than 1 m or 5 stairs; axial load to the head, for example, diving; high-speed motor vehicle collision; rollover motor accident; ejection from a motor vehicle; accident involving motorised recreational vehicles; bicycle collision) [27].

### Magnetic resonance imaging (MRI)

Magnetic resonance imaging is the best imaging modality and allows assessment for ligamentous damage, which may be missed on CT scanning. It is particularly useful in the obtunded or non-verbal child and when the cervical spine cannot be cleared within 72 h [26]. However, MRI is time-consuming and may not be appropriate in the unstable patient during the initial resuscitation phase. It is also costly and may not available out of hours, necessitating patient transfer to a tertiary centre. Younger children may also need to be anaesthetised to lie still for the duration of the scan and prevent motion artefact. The age at which a child can tolerate an MRI without an anaesthetic depends on a number of factors including: maturity, injury severity, radiographic staff experience with children, time in the scanner and MRI bore size. It also depends on whether it is just the cervical spine that is being imaged, or whether head imaging is also required. Nonetheless, it remains the gold standard for identifying cervical spine injuries.

## Pitfalls in imaging of the paediatric cervical spine

Normal variants in imaging of the child’s cervical spine may be confused for pathology. Moreover, there are key differences to assessment of cervical spine imaging in the adult patient. Although beyond the scope of the article to review all of these differences in detail, we highlight some of the important features. A good understanding of the embryology and paediatric cervical spine anatomy is necessary and early consultation with a specialist radiologist is a vital part of management of these children.

Prevertebral soft tissue thickening indicates injury in adults, but may occur during expiration or flexion of the neck in children and is especially prominent in the crying child [28]. If repeat radiography when the child settles is impossible then CT may be necessary.

Loss of cervical lordosis is an often-normal variant in children but may also result from cervical musculature spasm. The ossified portions of the cervical vertebrae are more spherical at birth and with age develop into their rectangular adult shape. Thus, young children may have a normal ‘wedge-appearance’ mimicking anterior wedge fractures seen in adults [29].

Moreover, measurements used for radiological diagnosis in adults are different in children due to anatomical differences described above. For example, the atlantodental interval (ADI), used to determine atlanto-occipital dislocation, is > 3 mm in adults and > 5 mm in children.

**Fig. 1:**
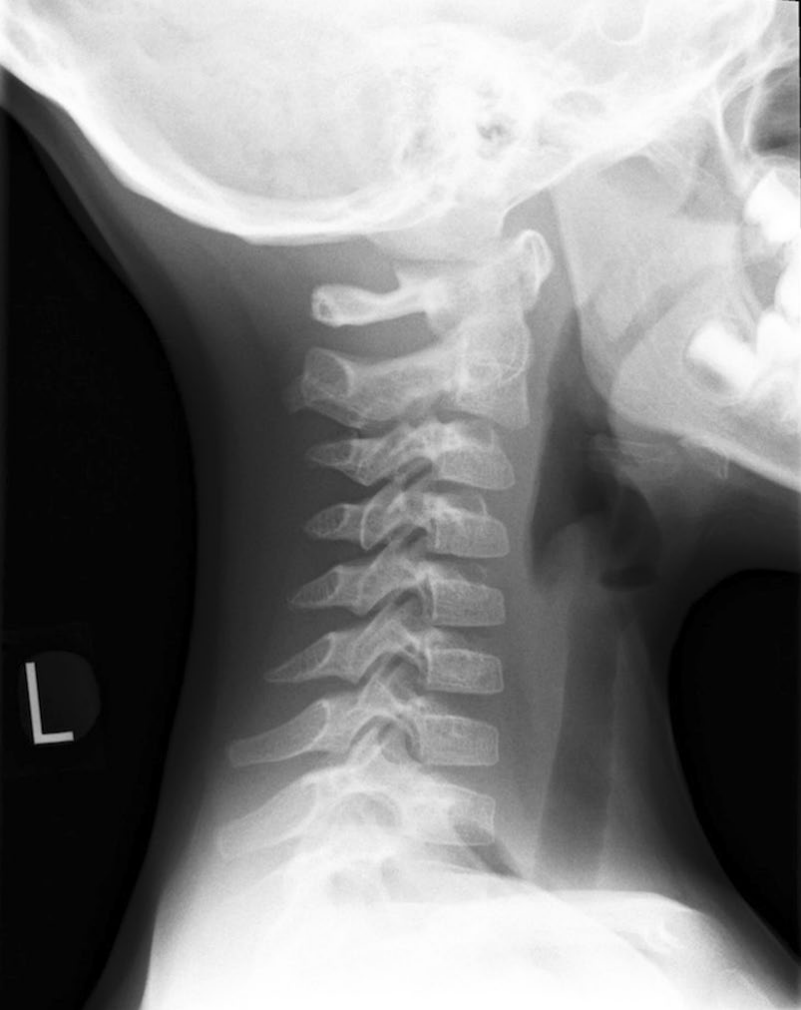
X-ray demonstrating C2 on C3 pseudosubluxation

Pseudosubluxation (especially at C2–3) may be noted on imaging due to the elasticity of the cervical spine (Fig. 1). The amount of subluxation is usually < 2 mm. The spinolaminar line should not be disrupted. Also the Swischuk line (drawn from the anterior aspect of the posterior arches of C1 down to C3) should be assessed. The line is usually < 1 mm anterior to the posterior arch of C2 and if the line is > 2 mm from that anterior aspect of the arch of C2 then pseudosubluxation is unlikely and assessment for dislocation should be performed (MRI is particularly useful here in determining ligamentous injury) [26].

**Fig. 2:**
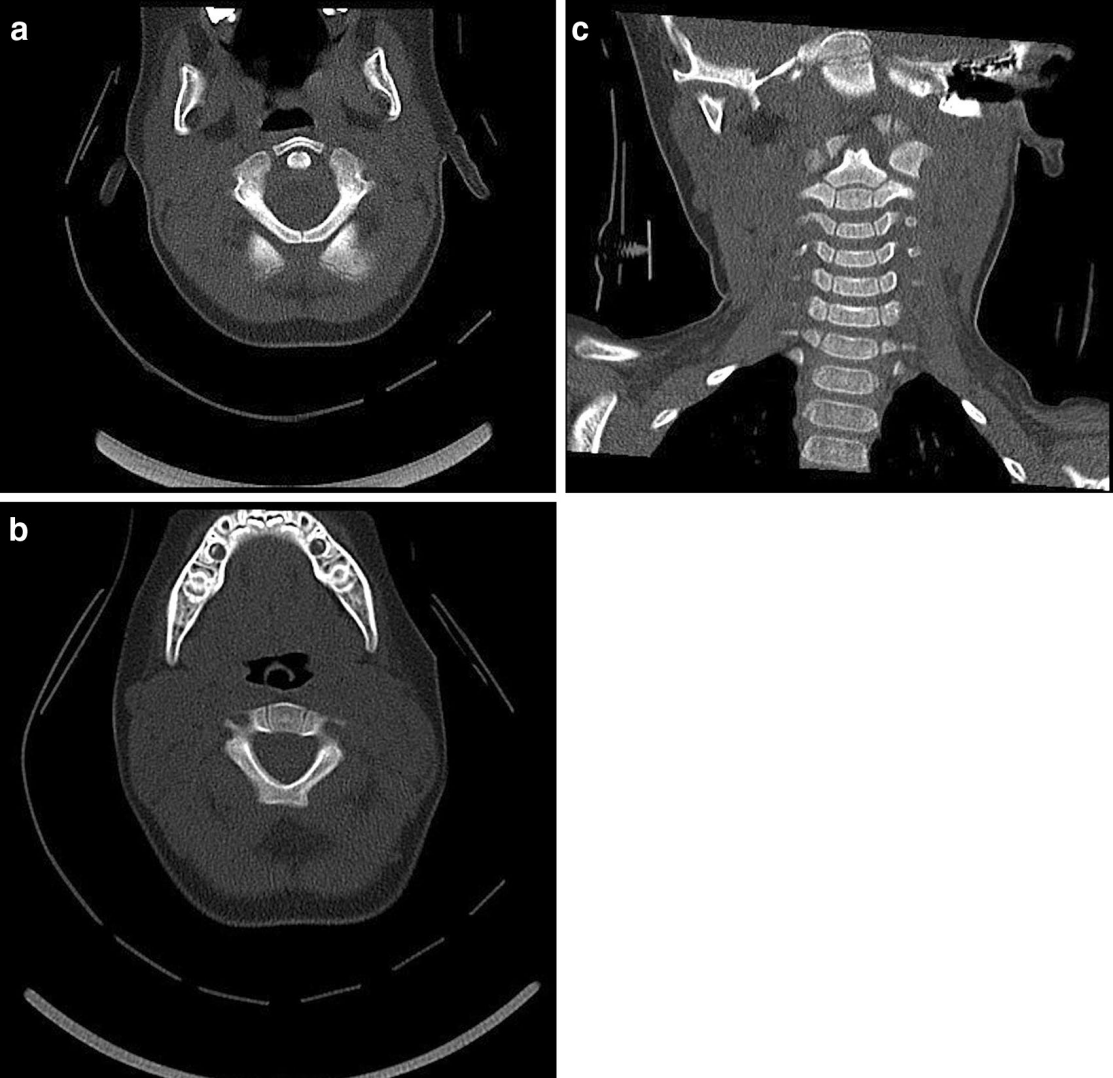
CT images demonstrating synchondroses of: **a** C1 on axial view; **b** C2 on axial view; and **c** the subaxial spine on coronal view

Synchondroses (the temporary epiphyseal growth plates that allow development of bony structures between ossification centres) may be mistaken for fractures on imaging (Fig. 2a–c). The location and appearance of synchondroses are predictable, and in most cases are symmetrical. It can be helpful to review imaging from a patient of a similar age to help determine normal anatomy. However, fractures may occur through synchondroses. When they do, they tend to fuse well and reduction and external immobilisation is the recommended treatment. Internal surgical fixation is attempted only if the fracture persists despite conservative treatment.

## Specific cervical spine injury patterns

### Atlanto-occipital dislocation (AOD)

This highly unstable pattern of injury is caused by high mechanical forces mostly seen during motor vehicle accidents [30]. Historically, it was thought that AOD was very infrequent and that those patients with AOD died at the scene due to the severity of this injury. However, more and more patients are surviving with this injury pattern due to improved awareness and initial care.

AOD is three times more common in children than adults due to the relatively large head and other anatomical differences [31, 32]. The joint capsule and surrounding ligaments at this articulation are looser in younger children. Moreover, the C1 arch is small, and the overlying foramen magnum is large and so translation of the occipital condyle across the lateral masses of C1 occurs with trauma and may lead to AOD [31, 32].

Neurological impairment is common, with 80% of patients having an abnormal neurological examination at the time of presentation [33]. However, difficulty arises as patients may present with subtle symptoms or may even be asymptomatic [31, 32]. High cervical cord injuries can lead to flaccid paralysis, sensory abnormalities, respiratory insufficiency/arrest, priapism, bowel/bladder incontinence and neurogenic shock.

**Fig. 3:**
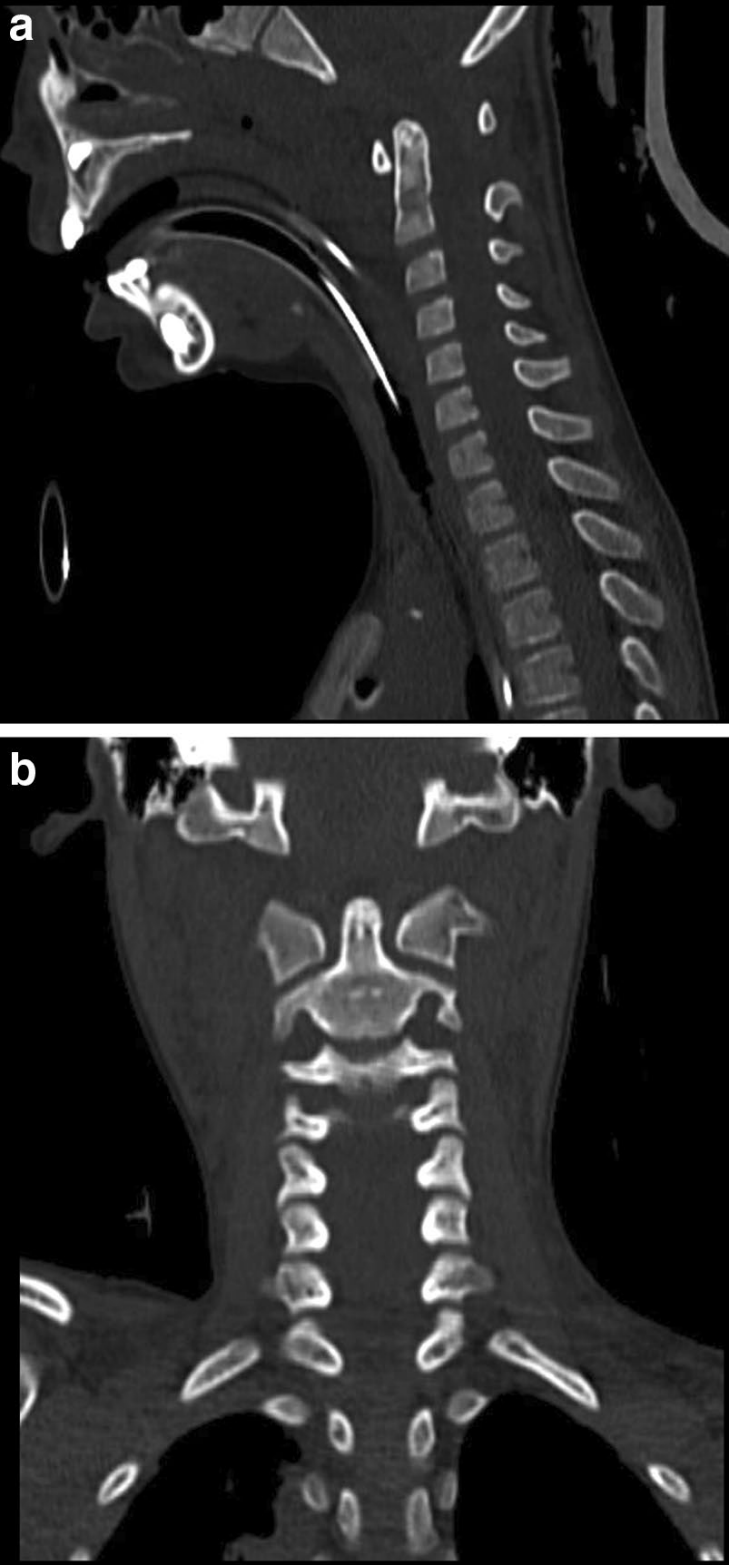
CT showing atlanto-occipital dislocation: **a** coronal; and **b** sagittal. Case courtesy of Dr Yair Glick, Radiopaedia.org, rID: 52910

Untreated, 54% of patients with AOD go on to develop permanent neurological deficit and 15% die [34]. Early stabilisation prevents associated morbidity and mortality [32]. CT imaging should be the initial imaging modality in the context of trauma as it will delineate any bony injury and other injuries sustained in the high-impact mechanism of injury (Fig. 3a, b). MRI scan should be performed subsequently to detect soft tissue/ligamentous injury, to further evaluate any neurological deficit and to plan treatment of the unstable injury. If the patient is unconscious and clinical evaluation is not possible then an MRI should be done within 48 h [35]. Horn et al. assessed outcomes of 33 patients with AOD and stratified patients into two groups based on imaging (Table 2) [36].

**Table 2:** Horn’s classification of AOD

Grade	CT findings	MRI findings
1	Normal	Moderately abnormal findings (e.g. high signal in the joint/ligaments)
2	Abnormal	Grossly abnormal findings in the joint/ligaments

Expert opinion regarding the management of AOD is contentious, largely due to a paucity of evidence as a result of its rarity. In grade 2 injuries, there is disruption of the normal ligamentous architecture of the occipito-cervical articulation, and external fixation will most likely not achieve healing. Thus, internal fixation, by occipito-cervical fixation and fusion, is generally accepted as being mandatory to achieve stability. If this is not possible initially, then a halo device can be applied in the interim. This initial external fixation prevents potential neurological worsening secondary to instability. Traction should be avoided during application of the halo device as it has been associated with a 10% risk of neurological deterioration [34]. Grade 2 injuries require post-operative imaging (usually CT) to assess for position of metalwork applied during internal fixation and to assess for any potential complications. In cases of grade 1 AOD, a trial period of 12 weeks of external orthosis with a Halo vest can be sufficient to allow healing. Following 12 weeks of immobilisation, grade 1 injuries should undergo repeat imaging to determine whether it is safe to trial removal of the external orthosis. Dynamic flexion/extension imaging is useful in ensuring no gross instability upon removal of immobilisation. Some children may require surgical fixation if satisfactory fusion is not achieved with conservative management.

### Atlanto-axial dislocation

This involves disarticulation between C1 and C2 (the atlanto-axial joint), leading to instability. The atlanto-axial joint is a complex structure comprised of three articulations: paired lateral joints that connect the lateral masses of C1–2 and a medial joint connecting the anterior and posterior aspect of the odontoid process (dens) of C2 to the anterior arch and the transverse atlantal ligament (TAL) of C1, respectively. The fibrous capsules of these joints are thin and thus almost the entirety of the atlanto-axial joint complex is supported by surrounding ligaments. The TAL runs from one lateral mass of the atlas across the posterior aspect of the odontoid peg (dens) to the contralateral lateral mass of the atlas. The ligament prevents anterior dislocation. Alar ligaments provide additional support by tethering the odontoid peg to the occiput. Trauma may result in damage to the TAL and thus dislocation of the C1–2 joint.

Whilst CT imaging may suggest disruption of the TAL by demonstration of bony injury at insertion sites on the lateral masses of C1, MRI is necessary to determine the integrity of the TAL. If the TAL itself is torn then surgical fixation is necessary as healing is unlikely. More commonly, if the lateral mass of C1 is fractured and the TAL is thus disrupted from the body of C1 a halo can be fitted in the first instance as 74% of these injuries heal without need for surgical intervention [37, 38].

### Axial fractures

Pure odontoid fractures are different in children compared to adults. The developing C2 vertebra consists of five ossification centres separated by six synchondroses. These synchondroses close between the ages of 7 and 13.5 years [39]. In young children, odontoid peg fractures usually occur through the synchondrosis between the C2 body and the peg. Synchondrosal fractures have been classified based on their pattern by Rusin et al. and usually heal well with external fixation [39]. Once the synchondroses have closed, injuries can be classified as per adults, using the Anderson and D’Alonzo classification [2].

The Anderson–D’Alonzo classification [1974] divided odontoid peg fractures into three types: odontoid tip fracture (type I), base of odontoid tip fracture (type II) and a fracture extending through the body of C2 and disrupting the odontoid from its base (type III). Type II fractures can lead to atlanto-axial dislocation [40]. Treatment is with the halo vest if type I or III, as these are relatively stable. Type II fractures are treated in the same way if the dens is displaced less than 5 mm. If more displaced, surgical intervention may be necessary either by odontoid screw fixation or fusion of the atlanto-axial joint.

Neurological deficit after odontoid fracture is uncommon [2]. If they occur in isolation, odontoid peg fractures heal well when managed conservatively; however, if concomitant atlanto-axial dislocation is present then internal fixation is necessary [2].

**Fig. 4:**
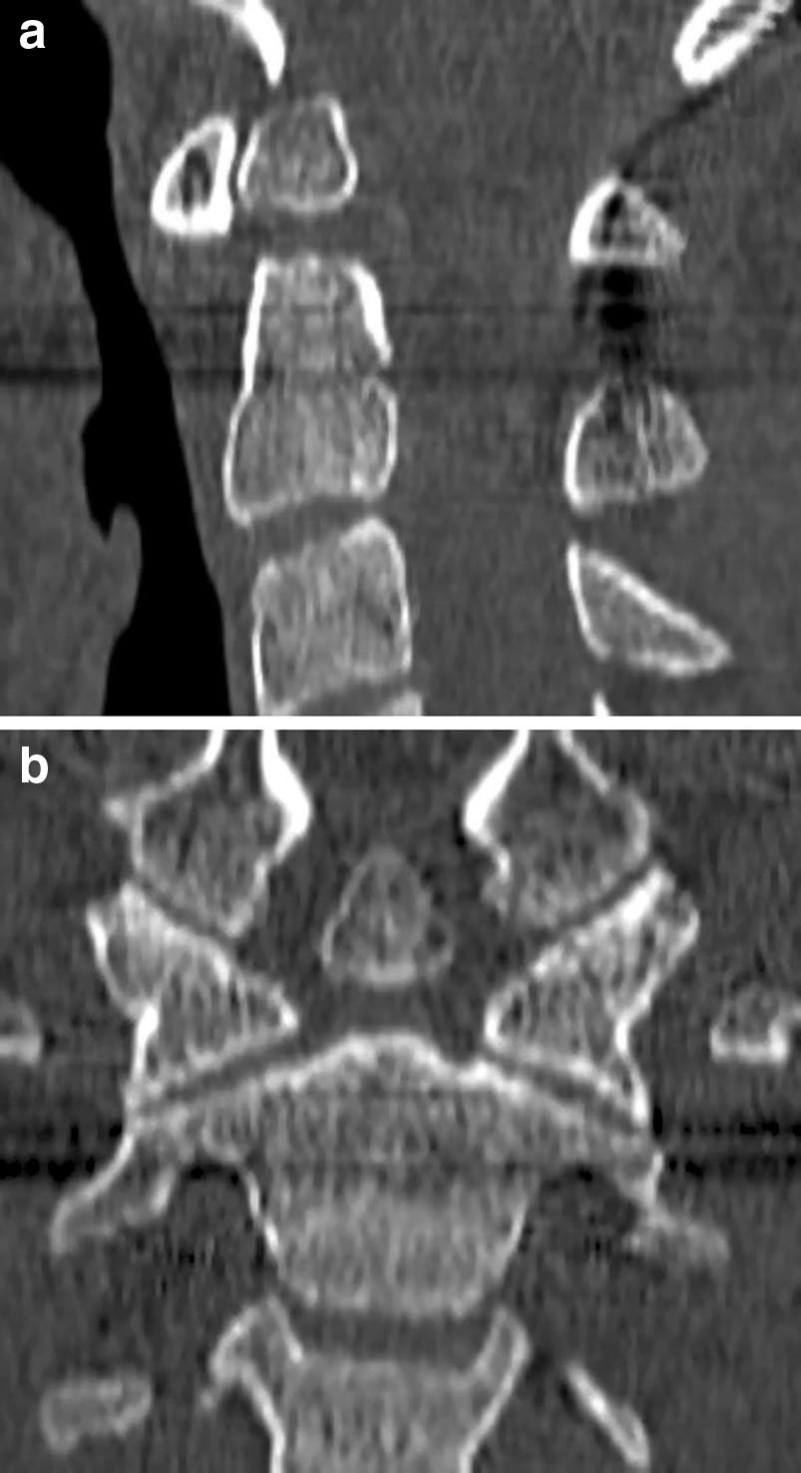
CT example of orthotopic Os Odontoideum. **a** Sagittal; and **b** coronal view. Case courtesy of Dr Roberto Schubert, Radiopaedia.org, rID: 14258

Os odontoideum is a rare abnormality characterised by a separation of a portion of the odontoid process from the remaining part associated with the body of the axis (Fig. 4a, b). It may in fact be caused by an occult traumatic fracture in childhood. It can be termed orthotopic if in the usual position of the normal odontoid peg, or dystopic if abnormally located [41]. Trauma can lead to compression of the os odontoideum against the spinal cord and this condition predisposes to atlanto-axial dislocation due to the truncated odontoid peg, over which the transverse ligament may slip during trauma [40]. Operative intervention is indicated in the presence of neurological signs/symptoms, pain, if there is progressive instability of the atlanto-axial complex or if there is > 5 mm translational instability in the antero-posterior direction [41].

### Atlanto-axial rotary fixation (AARF)

Atlanto-axial rotary fixation can present with complete limitation in the ability to rotate the neck or with the so-called ‘cock-robin’ deformity (fixed rotation of the neck and contralateral flexion). AARF may occur in the context of minor trauma, following an upper respiratory tract infection or spontaneously. The shallow articulation of the C1–2 lateral masses and the highly elastic supporting ligamentous structures predispose children to this injury. Typically, the ipsilateral sternocleidomastoid is in spasm, which contrasts to the contralateral spasm seen in torticollis that is not due to AARF (e.g. acquired benign paroxysmal torticollis, cervical lymphadenitis, cervical spine/cord tumours, and posterior fossa tumours).

**Fig. 5:**
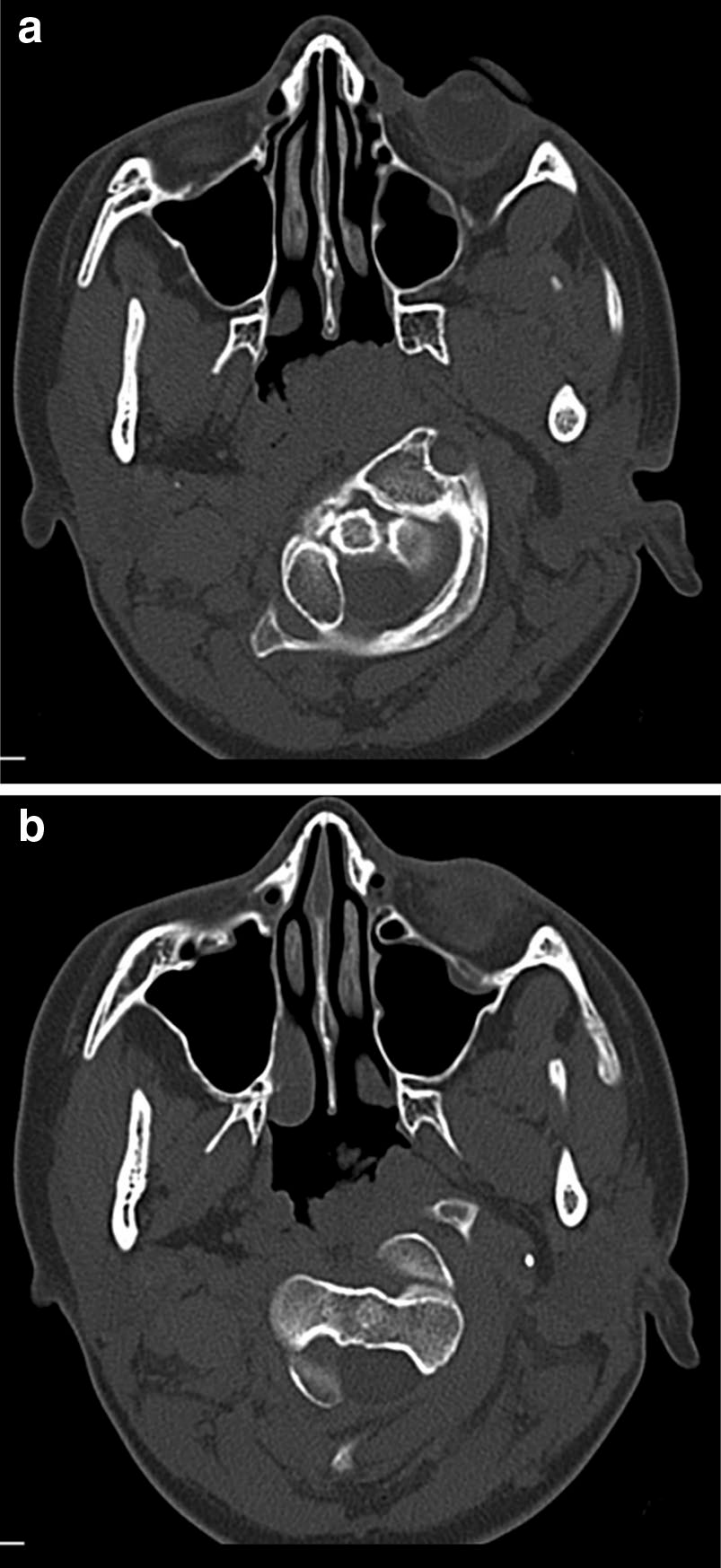
**a, b** Axial CT images demonstrating rotatory subluxation of C1 on C2. The left lateral mass is located anterior and lateral to its normal position and the right lateral mass is located posteriorly and medially

Traumatic mechanisms of injury are more likely to cause damage to the ligamentous support system of the C1–C2 complex, with a greater degree of anterior/posterior dislocation and thus greater propensity towards neurological deficit. Occipital neuralgia may also be caused as the greater occipital nerve is impinged by the subluxed vertebrae. Plain radiography is difficult to assess due to the abnormal neck posture. Lateral view may show one lateral mass to be more anterior to its counterpart [42]. The odontoid view may show asymmetry of the lateral masses but achieving this view is technically difficult [42]. CT is preferable as it is the most sensitive investigation for demonstration of rotation of the atlas over the axis and as it may show associated fractures that are difficult to appreciate on the plain radiographs (Fig. 5a, b) [42]. Assessment of the atlanto-occipital joint is also achieved and can demonstrate instability here that potentiates the instability at C1-C2 [42]. MRI is useful in assessing for ligamentous injury and spinal cord contusions but is not as sensitive as CT scanning for initial diagnosis. Spontaneous reduction is not uncommon and patients should be managed conservatively with gentle reduction followed by external immobilisation, as functional outcome is excellent [43, 44]. Internal posterior fixation of C1-2 is reserved for those with neurological deficit, significant instability with ligamentous injury, recurrent dislocations or those that respond poorly to conservative management [23].

### Subaxial injuries

Injuries to the subaxial vertebra can include vertebral body fractures, subluxations, dislocation of facet joints and fractures of the laminar/pedicular/spinous processes. Subaxial injuries in the paediatric population are predominantly seen in older children. The injury patterns in children are similar to those of adults because after the age of eight, the subaxial cervical spine is well developed and resembles that of an adult. In these patients, the most commonly affected region is the C5–7 because the fulcrum has descended from the higher position it has in infancy [2, 45]. Treatment of such fractures is similar to adults and may involve use of a halo device or internal fixation. Subaxial fractures in younger children may often be managed with a cervical collar alone, depending on injury type, stability and whether neurological deficit is present [45].

## SCIWORA

**Fig. 6:**
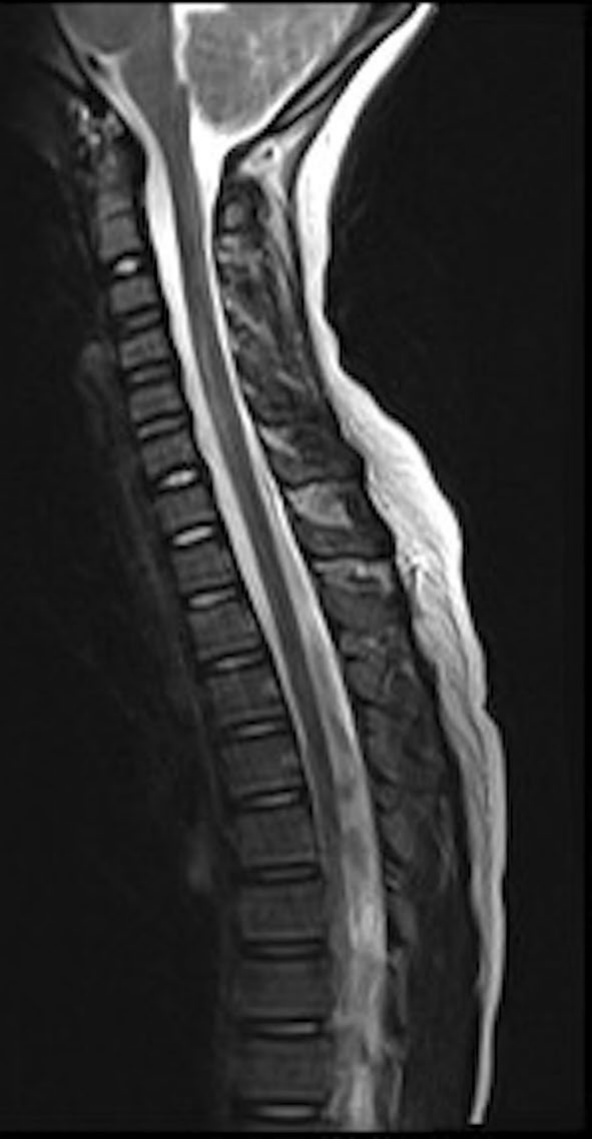
Sagittal T2-weighted MR image showing SCIWORA. Note the hyperintensity of cord oedema following trauma to the cervical spine

This syndrome was first described by Pang et al. in [6] as “objective signs of myelopathy resulting from trauma, with no evidence of ligamentous injury or fractures on plain radiographs or tomographic studies”. Advances in radiology have since found that upon performing MRI, soft tissue injuries are identified, including: ligamentous damage, disc herniation, small end-plate fractures and splitting of the hypervascular growth zone from the vertebral end-plate. Some patients with features of SCIWORA have very subtle findings on MRI and indeed the extent of injury on MRI often correlates with prognosis (Fig. 6).

SCIWORA is more common in younger children and tends to occur in the upper cervical spine. After the age of 9 it is rarely noted in the upper cervical spine but remains a cause of injury in the lower cervical spinal cord up to the age of 16 [5]. Prognosis is better in the older children with lower cervical spine involvement [5].

The mechanism involved in this injury pattern is flexion–extension. Flexion can rupture the anterior longitudinal ligament which allows intervertebral displacement that reduces spontaneously due to the elasticity of the structures in children. As such, no abnormality is initially picked up on radiography or computed tomography. Extension causes buckling of the ligamentum flavum, which in turn compresses the spinal cord. The pattern of subsequent spinal cord injury may be complete transection, a partial cord syndrome, a central cord syndrome or a Brown–Séquard syndrome. One systematic review of all paediatric cases of SCIWORA found that of 433 reported cases, 87.2% affected the cervical region [7]. Of these, 263 had sufficient information to determine presenting ASIA score; 69.6% were ASIA D, 7.6% ASIA C, 5.3% ASIA B and 17.5% ASIA A. Those presenting with ASIA A showed no recovery. Those presenting with ASIA B had a 21.4% rate of complete recovery, those presenting with ASIA C had a 35% rate of complete recovery and those presenting with ASIA D had a 97.3% rate of complete recovery [7].

SCIWORA is traditionally treated by external immobilisation for up to 3 months, to allow the tissues to heal. Some authors advocate a shorter period of external immobilisation in cases of SCIWORA that do not involve neural tissue damage on MRI [5]. Children should be encouraged to refrain from activities that may predispose to further trauma to the cervical spine for at least 6 months [46]. On follow-up, repeat imaging should be performed at 3 months or prior to the removal of the cervical collar, This is done by trained practitioners using flexion–extension radiographs to ensure that there are no features of late instability. Repeat magnetic resonance imaging is only necessary in the event of a novel or evolving neurological deficit during flexion/extension imaging or the following removal of the collar.

## Definitive management of injury

Owing to its intrinsic elasticity, the cervical spine of a child is more difficult to immobilise than that of an adult. External fixation may be achieved to some degree with a rigid collar. However, some rotation still occurs and full stability, especially in the upper and middle cervical spines, is not achieved. The halo device for external fixation is useful in adults, but in children, the same approach is often problematic. The shallower nature of the child’s skull may make pin fixation more prone to complications (e.g. pin site infection, pin loosening, dural penetration, supra-orbital nerve injury and scarring) [47]. Furthermore, the biopsychosocial effect of being confined within the halo system cannot be underestimated. Importantly, the device impairs the child’s ability to recover physically and mentally from the injury. The Minerva body jacket obviates the use of pins, negating these potential complications. It has been shown to be as effective as the halo in stabilisation of the mid to lower portion of the cervical spine but is not as effective in stabilising upper cervical spine injuries [48].

Internal (surgical) fixation is necessary in some cases to provide stability and to protect the spinal cord from damage. Indications include non-reducible deformities, unstable injuries requiring stabilisation, progressive deformity, and decompression of neural structures [5]. Whilst the principles of internal fixation remain the same in adults as in children, there are specific considerations. The potential growth of the spine needs to be taken into consideration as instrumentation can inhibit future growth and affect the curvature of the spine. Growth is driven at the vertebral body endplates in the anterior column of the cervical spine. By 10 years, the cervical vertebrae are near adult size and surgical intervention is less likely to have a significant impact on children as they continue to grow. Post-operatively, children must be carefully followed up to assess for any complications. Long-term follow-up is vital in the younger patients whose spine continues to grow as instrumentation may lead to deformity and hinder normal development.

### Associated cranio-cervical arterial injuries

Cranio-cervical arterial dissection (CCAD) can occur in conjunction with injuries sustained to the cervical spine. Although children lack the atherosclerotic changes seen in adults that make vessels vulnerable to CCAD (especially at the carotid bifurcation); the greater head:body proportion, weaker neck musculature and greater reliance on ligamentous (rather than bony) structures predisposes children to a greater risk of post-traumatic CCAD [49]. Indeed, the most mobile segments of the arteries in the neck are at greatest risk and thus CCAD is most often seen in the distal cervical internal carotid artery (just as it enters the skull base) or in the proximal segments of the vertebral arteries. A tear can occur through the intima with sub-intimal haematoma causing stenosis as the false lumen of the dissection encroaches upon the diameter of the true lumen. A breech of the vessel wall underneath the adventitial layer may also lead to development of a pseudoaneurysm. Emboli may be thrown off as a consequence of CCAD/pseudoaneurysm and can cause immediate or late onset transient ischaemic attacks or ischaemic strokes. A Horner’s syndrome may also be noted if the sympathetic chain is damaged.

Pandley et al. assessed a series of 42 patients under 25 years old with CCAD and found 81% were secondary to trauma [50]. Cervical spine injuries were noted in 23.5% [50]. CT and MR angiography are both excellent non-invasive methods of assessment of underlying CCAD, with MR being preferable in terms of reducing the risks associated with ionising radiation but CTA being much more rapid in the context of a potentially unstable trauma patient. Radiological characteristics of cranio-cervical arterial injuries include vessel occlusion, stenosis, intimal flaps/double lumen sign and pseudoaneurysm formation.

Management strategies are controversial and complex. There is no high-quality evidence to direct therapy, thus management at present mirrors that of the adult patient. Typically, in asymptomatic cases of CCAD, medical treatment is with an antithrombotic, either aspirin or clopidogrel for 3–6 months as first-line therapy [51]. Further anticoagulation (usually low molecular weight heparin or warfarin) may be considered if symptoms progress. The American Heart Association recommends treating ischaemic strokes secondary to extra-cranial CCAD with 3–6 months of low molecular weight heparin or warfarin [52]. However, these medical therapies increase the risk of *de novo* haemorrhage and so concomitant injuries (especially intracranial) may prevent such escalation of medical therapy and thus therapy should be tailored to the patient on a case-by-case basis [53].

Endovascular management of pseudoaneurysms and dissections is evolving. There have been case reports of successful treatment of pseudoaneurysms in both blunt and penetrating trauma to the paediatric cervical spine [51, 54]. Management in these cases is initially medical, with follow-up imaging (consisting of CTA, MRA and ultrasound) showing enlargement of the pseudoaneurysm that prompted intervention [51, 54]. In the context of an enlarging pseudoaneurysm or progressive narrowing of the diameter of the stenotic vessel, endovascular stenting should be considered. There is no guidance as to the timing and manner of follow-up following diagnosis of CCAD, but ultrasound and MRA would be the safest modalities in children, with MRA giving much better image quality. In the stable patient in whom neurological examination can be performed, follow-up imaging should be done at 6–8 weeks, with the proviso that any new or progressive neurological deficit should be investigated immediately. In select cases in which there is concomitant traumatic brain injury and either the patient cannot be fully assessed clinically, or it is difficult to determine if a progression in symptoms is attributable to evolution of the cerebral injury or as a consequence of the CCAD, more expedient follow-up may be prudent (i.e. in the first 1–2 weeks). In such a scenario, CTA may be preferable when dealing with a potentially unstable patient being transferred from an intensive care setting.

## Conclusion

Injury to the cervical spine can have devastating consequences for a child. Successful management of these children requires an understanding of the differences between the adult and paediatric anatomy and the specific patterns of pathologies that frequently occur in children. Many controversies remain and high quality evidence is required to determine best practice in such cases.

## References

[1] Patel JC, Tepas JJ, Mollitt DL, Pieper P (2001). Pediatric cervical spine injuries: defining the disease. J Pediatr Surg.

[2] Baumann F, Ernstberger T, Neumann C, Nerlich M, Schroeder GD, Vaccaro AR (2015). Pediatric cervical spine injuries: a rare but challenging entity. J Spinal Disord Tech.

[3] Vogel LC (1997). Unique management needs of pediatric spinal cord injury patients: etiology and pathophysiology. J Spinal Cord Med.

[4] Huisman TA, Phelps T, Bosemani T, Tekes A, Poretti A (2015). Parturitional injury of the head and neck. J Neuroimaging.

[5] Mortazavi M, Gore PA, Chang S, Tubbs RS, Theodore N (2011). Pediatric cervical spine injuries: a comprehensive review. Childs Nerv Syst.

[6] Pang D, Wilberger JE (1982). Jr. Spinal cord injury without radiographic abnormalities in children. J Neurosurg.

[7] Carroll T, Smith CD, Liu X, Bonaventura B, Mann N, Liu J (2015). Spinal cord injuries without radiologic abnormality in children: a systematic review. Spinal Cord.

[8] Huerta C, Griffith R, Joyce SM (1987). Cervical spine stabilization in pediatric patients: evaluation of current techniques. Ann Emerg Med.

[9] Group ALS (2016). Advanced paediatric life support: a practical approach to emergencies (APLS).

[10] National Institute for Health and Care Excellence. Spinal injury: assessment and initial management. (NICE guideline 41). 2014. https://www.nice.org.uk/guidance/ng41. 26913323

[11] Dickman CA, Papadopoulos SM, Sonntag VK, Spetzler RF, Rekate HL, Drabier J (1993). Traumatic occipitoatlantal dislocations. J Spinal Disord.

[12] Herzenberg JE, Hensinger RN, Dedrick DK, Phillips WA (1989). Emergency transport and positioning of young children who have an injury of the cervical spine. The standard backboard may be hazardous. J Bone Joint Surg Am.

[13] Bracken MB, Shepard MJ, Collins WF, Holford TR, Young W, Baskin DS (1990). A randomized, controlled trial of methylprednisolone or naloxone in the treatment of acute spinal-cord injury. Results of the Second National Acute Spinal Cord Injury Study. N Engl J Med.

[14] Bracken MB, Shepard MJ, Holford TR, Leo-Summers L, Aldrich EF, Fazl M (1997). Administration of methylprednisolone for 24 or 48 hours or tirilazad mesylate for 48 hours in the treatment of acute spinal cord injury. Results of the Third National Acute Spinal Cord Injury Randomized Controlled Trial. National Acute Spinal Cord Injury Study. JAMA.

[15] Pettiford JN, Bikhchandani J, Ostlie DJ, St Peter SD, Sharp RJ, Juang D (2012). A review: the role of high dose methylprednisolone in spinal cord trauma in children. Pediatr Surg Int.

[16] Hurlbert RJ, Hadley MN, Walters BC, Aarabi B, Dhall SS, Gelb DE (2013). Pharmacological therapy for acute spinal cord injury. Neurosurgery.

[17] Fehlings MG, Wilson JR, Cho N (2014). Methylprednisolone for the treatment of acute spinal cord injury: counterpoint. Neurosurgery.

[18] Geisler FH, Coleman WP, Grieco G, Poonian D, Sygen Study G (2001). The Sygen multicenter acute spinal cord injury study. Spine (Phila Pa 1976).

[19] Ahuja CS, Martin AR, Fehlings M. Recent advances in managing a spinal cord injury secondary to trauma. F1000Res. 2016;5. 10.12688/f1000research.7586.1. 10.12688/f1000research.7586.1 PMC4890313 27303644

[20] Furlan JC, Noonan V, Cadotte DW, Fehlings MG (2011). Timing of decompressive surgery of spinal cord after traumatic spinal cord injury: an evidence-based examination of pre-clinical and clinical studies. J Neurotrauma.

[21] Viccellio P, Simon H, Pressman BD, Shah MN, Mower WR, Hoffman JR (2001). A prospective multicenter study of cervical spine injury in children. Pediatrics.

[22] Hoffman JR, Schriger DL, Mower W, Luo JS, Zucker M (1992). Low-risk criteria for cervical-spine radiography in blunt trauma: a prospective study. Ann Emerg Med.

[23] Rozzelle CJ, Aarabi B, Dhall SS, Gelb DE, Hurlbert RJ, Ryken TC (2013). Management of pediatric cervical spine and spinal cord injuries. Neurosurgery.

[24] Garton HJ, Hammer MR (2008). Detection of pediatric cervical spine injury. Neurosurgery.

[25] Buhs C, Cullen M, Klein M, Farmer D (2000). The pediatric trauma C-spine: is the ‘odontoid’ view necessary?. J Pediatr Surg.

[26] Booth TN (2012). Cervical spine evaluation in pediatric trauma. AJR Am J Roentgenol.

[27] National Institute for Health and Care Excellence. Head injury: triage, assessment, investigation and early management of head injury in infants, children and adults. (Clinical guideline 176). 2014. https://www.nice.org.uk/CG176. 25340248

[28] Vermess D, Rojas CA, Shaheen F, Roy P, Martinez CR (2012). Normal pediatric prevertebral soft-tissue thickness on MDCT. AJR Am J Roentgenol.

[29] Swischuk LE, Swischuk PN, John SD (1993). Wedging of C-3 in infants and children: usually a normal finding and not a fracture. Radiology.

[30] Marshall KW, Koch BL, Egelhoff JC (1998). Air bag-related deaths and serious injuries in children: injury patterns and imaging findings. AJNR Am J Neuroradiol.

[31] Garrett M, Consiglieri G, Kakarla UK, Chang SW, Dickman CA (2010). Occipitoatlantal dislocation. Neurosurgery.

[32] Hall GC, Kinsman MJ, Nazar RG, Hruska RT, Mansfield KJ, Boakye M (2015). Atlanto-occipital dislocation. World J Orthop.

[33] Harmanli O, Koyfman Y (1993). Traumatic atlanto-occipital dislocation with survival: a case report and review of the literature. Surg Neurol.

[34] Theodore N, Aarabi B, Dhall SS, Gelb DE, Hurlbert RJ, Rozzelle CJ (2013). The diagnosis and management of traumatic atlanto-occipital dislocation injuries. Neurosurgery.

[35] Riascos R, Bonfante E, Cotes C, Guirguis M, Hakimelahi R, West C (2015). Imaging of Atlanto-occipital and atlantoaxial traumatic injuries: what the radiologist needs to know. Radiographics.

[36] Horn EM, Feiz-Erfan I, Lekovic GP, Dickman CA, Sonntag VK, Theodore N (2007). Survivors of occipitoatlantal dislocation injuries: imaging and clinical correlates. J Neurosurg Spine.

[37] Dickman CA, Greene KA, Sonntag VK (1996). Injuries involving the transverse atlantal ligament: classification and treatment guidelines based upon experience with 39 injuries. Neurosurgery.

[38] Lo PA, Drake JM, Hedden D, Narotam P, Dirks PB (2002). Avulsion transverse ligament injuries in children: successful treatment with nonoperative management. Report of three cases. J Neurosurg.

[39] Rusin JA, Ruess L, Daulton RS (2015). New C2 synchondrosal fracture classification system. Pediatr Radiol.

[40] Yang SY, Boniello AJ, Poorman CE, Chang AL, Wang S, Passias PG (2014). A review of the diagnosis and treatment of atlantoaxial dislocations. Global Spine J.

[41] Robson KA (2011). Os odontoideum: rare cervical lesion. West J Emerg Med.

[42] Roche CJ, O’Malley M, Dorgan JC, Carty HM (2001). A pictorial review of atlanto-axial rotatory fixation: key points for the radiologist. Clin Radiol.

[43] Pang D, Li V (2005). Atlantoaxial rotatory fixation: part 3-a prospective study of the clinical manifestation, diagnosis, management, and outcome of children with atlantoaxial rotatory fixation. Neurosurgery.

[44] Glotzbecker MP, Wasser AM, Hresko MT, Karlin LI, Emans JB, Hedequist DJ (2014). Efficacy of nonfusion treatment for subacute and chronic atlanto-axial rotatory fixation in children. J Pediatr Orthop.

[45] Murphy RF, Davidson AR, Kelly DM, Warner WC, Sawyer JR (2015). Subaxial cervical spine injuries in children and adolescents. J Pediatr Orthop.

[46] Szwedowski D, Walecki J (2014). Spinal cord injury without radiographic abnormality (SCIWORA)—clinical and radiological aspects. Pol J Radiol.

[47] Dormans JP, Criscitiello AA, Drummond DS, Davidson RS (1995). Complications in children managed with immobilization in a halo vest. J Bone Joint Surg Am.

[48] Benzel EC, Larson SJ, Kerk JJ, Millington PJ, Novak SM, Falkner RH (1992). The thermoplastic Minerva body jacket: a clinical comparison with other cervical spine splinting techniques. J Spinal Disord.

[49] Orman G, Tekes A, Poretti A, Robertson C, Huisman TA (2014). Posttraumatic carotid artery dissection in children: not to be missed!. J Neuroimaging.

[50] Pandey AS, Hill E, Al-Holou WN, Gemmete JJ, Chaudhary N, Thompson BG (2015). Management of pediatric craniocervical arterial dissections. Childs Nerv Syst.

[51] Wang A, Santarelli JG, Stiefel MF (2016). Traumatic cervical internal carotid artery pseudoaneurysm in a child refractory to initial endovascular treatment: case report and technical considerations. Childs Nerv Syst.

[52] Roach ES, Golomb MR, Adams R, Biller J, Daniels S, Deveber G (2008). Management of stroke in infants and children: a scientific statement from a Special Writing Group of the American Heart Association Stroke Council and the Council on Cardiovascular Disease in the Young. Stroke.

[53] Harrigan MR, Hadley MN, Dhall SS, Walters BC, Aarabi B, Gelb DE (2013). Management of vertebral artery injuries following non-penetrating cervical trauma. Neurosurgery.

[54] Lucas ONH, Davies JM, Reynold R, Bass KD (2017). Endovascular treatment of a carotid artery pseudoaneurysm due to penetrating trauma in a pediatric patient. J Pediatr Surg Case Rep.

